# High Levels of CXCL8 and Low Levels of CXCL9 and CXCL10 in Women with Maternal RhD Alloimmunization

**DOI:** 10.3389/fimmu.2017.00700

**Published:** 2017-07-03

**Authors:** Juliana Araújo de Carvalho Schettini, Thomás Virgílio Gomes, Alexandra Karla Santos Barreto, Claudeir Dias da Silva Júnior, Marina da Matta, Isabela Cristina Neiva Coutinho, Maria do Carmo Valgueiro Costa de Oliveira, Leuridan Cavalcante Torres

**Affiliations:** ^1^Translational Research Laboratory, Instituto de Medicina Integral Prof. Fernando Figueira (IMIP), Recife, Brazil; ^2^Department of Obstetrics and Gynecology, Instituto de Medicina Integral Prof. Fernando Figueira (IMIP), Recife, Brazil; ^3^Hematology and Hemotherapy Foundation of Pernambuco (HEMOPE), Recife, Brazil

**Keywords:** chemokines, pregnancy, inflammation, flow cytometry, Rh(o) antigen, genotyping techniques, hemolytic disease of the fetus and newborn

## Abstract

Maternal RhD alloimmunization is an inflammatory response against protein antigens in fetal red blood cells (RBC). However, not all women become alloimmunized when exposed to RhD^+^ fetal RBC. Thus, this study aimed to evaluate levels of inflammatory chemokines in RhD^−^ pregnant women with erythrocyte alloimmunization. CXCL8, CXCL9, CCL5, and CXCL10 levels were determined from cell culture supernatants by flow cytometry in 46 (30 non-alloimmunized RhD^−^ and 16 previously alloimmunized RhD^−^) pregnant women. CXCL8 levels were significantly higher (*P* < 0.004), and CXCL9 (*P* < 0.008) and CXCL10 (*P* < 0.003) levels were significantly lower in alloimmunized pregnant women. No significant difference in CCL5 levels was detected between the groups. Fetal RHD genotyping was performed in the alloimmunized RhD^−^ group by real-time PCR. Anti-D alloantibody was detected in 10 mothers and anti-D and -C in six mothers. Twelve fetuses were RHD positive and four were RHD negative. Further studies of serum chemokines and placenta tissue could provide a better understanding of the cells involved in the pathogenesis of maternal erythrocyte alloimmunization.

## Introduction

Chemokines are essential to stimulate chemotaxis of leukocytes and initiate inflammatory responses (1, 2). In pregnancy, chemokines are potent mediators of embryogenesis and neoangiogenesis and important for the recruitment of macrophages and NK, dendritic, and T cells to maternal decidua (1, 2). There is increasing evidence suggesting a relationship between maternal inflammatory status and disease pathogenesis in pregnancy (3, 4). However, it remains unclear why some pregnant women do not become alloimmunized during pregnancy even when exposed to high amounts of RhD-positive fetal red blood cells (RBC). Thus, analysis of inflammatory chemokines could provide a better understanding of the pathogenesis of maternal erythrocyte alloimmunization.

Maternal RhD alloimmunization is an immune response against protein antigens in fetal RBC (5). Studies in murine models of RBC alloimmunization showed increased RBC antibody (RBCA) production associated with artificially induced inflammation (6). Some diseases with excessive immune system activation such as sickle cell anemia (7) and thalassemia (8) and also transfusion of blood cell components, cell, tissue, and organ transplantation are associated with a high risk of RBC alloimmunization (9).

However, studies relating human erythrocyte alloimmunization in pregnancy and inflammation are lacking. Thus, this study aimed to evaluate the levels of inflammatory chemokines in RhD^−^ pregnant women with erythrocyte alloimmunization.

## Materials and Methods

The study was conducted at the Pregnancy Outpatient Clinic and Anthony Hart Translational Research Laboratory of Professor Fernando Figueira Integrative Medicine Institute (IMIP), Recife, Brazil, between August 2013 and December 2014. The two laboratories operate under exploratory research principles. Clinical and laboratory protocols were approved by the IMIP Research Ethics Committee (no. 3437-13). Written informed consent was obtained from all participants.

### Subjects

All pregnant women were eligible if they intended on continuing antenatal visits at IMIP. Maternal RhD alloimmunization was determined by a positive indirect antigammaglobulin test (IAT^+^). Thirty RhD-negative pregnant women (RhD^−^/IAT^−^) and 16 alloimmunized RhD-negative (RhD^−^/IAT^+^) pregnant women were included. All RhD^−^/IAT^+^ women had became alloimmunized in previous pregnancies. Except for alloimmunized women, all were low-risk, second- and third-trimester single pregnancies. Gestational age was based on early ultrasound assessment. All participants provided a full detailed medical history and underwent complete general and obstetric examination. Screening for human immunodeficiency virus 1 and 2, hepatitis B and C, and human T lymphotropic virus type 1 and 2, and antinuclear antibody test was performed for all women.

The exclusion criteria were (i) family or personal history of autoimmune diseases and/or positive antinuclear antibody test, (ii) infections, (iii) history of bone marrow or organ transplant, (iv) *in vitro* fertilization, (v) personal history of malignancy, and (vi) primary and/or secondary immunodeficiency.

### RhD-Negative Pregnant Group (RhD^−^)

Alloimmunized (IAT^+^) and non-alloimmunized (IAT^−^) RhD^−^ pregnant women were followed up for all subsequent antenatal consults with the principal investigator until 60 days after birth. In each visit, obstetric and clinical information was collected and updated. *RHD* fetal genotyping was determined for RhD^−^/IAT^+^ mothers. For RhD^+^ fetuses, intensive antenatal monitoring for detection of fetal anemia was performed by obstetric color Doppler ultrasonography with assessment of middle cerebral artery peak systolic velocity (MCA-PSV). Cordocentesis was performed for the detection of fetal hydrops and/or anemia (MCA-PSV >1.5 multiples of the median). When anemia was present (fetal Hb <10 g/dL, hematocrit <30%), cordocentesis was followed by intrauterine transfusion (IUT) with irradiated, leukocyte-depleted and washed, type O, RhD^−^ RBC. Medical records were reviewed to retrieve birth, postpartum, and neonatal information. All RhD-negative IAT^−^ pregnant women receive Rh immune globulin following delivery of an RhD-positive fetus with direct antiglobulin test negative. Postnatal anti-D prophylaxis was given as soon as possible, not exceeding the first 72 h after delivery. Ante-natally, anti-D prophylaxis was given just following potential sensitizing events such as antepartum hemorrhage, invasive prenatal diagnosis, any abdominal trauma (direct/indirect, sharp/blunt, open/closed), after miscarriage, childbirth, and fetal death and after birth with RhD-positive newborns with direct antiglobulin test negative. Among 30 RhD-negative IAT^−^, about 23 were treated with Rh immune globulin after previous pregnancies (more than 2 years ago) and seven were nullipara.

### Blood Samples

Blood group typing data for the entire cohort were retrieved from medical records. From each participant, 4 mL of peripheral blood was collected into a BD Vacutainer^®^ EDTA anticoagulant tube (BD Biosciences, San Jose, CA, USA) and 9 mL was collected into a BD Vacutainer^®^ sodium heparin tube for cell culture and processed in less than 3 h. In addition, 4 mL of peripheral blood was collected from alloimmunized women into a BD Vacutainer^®^ sterile tube (without anticoagulant) for IAT, and an extended 11-cell panel was referred to the Hematology and Hemotherapy Foundation of Pernambuco (HEMOPE) for RBCA identification in less than 3 h. In a subsequent prenatal visit, 16 mL of peripheral venous blood was collected into a BD Vacutainer^®^ K2 EDTA tube for fetal *RHD* genotyping in maternal plasma.

### Peripheral Blood Mononuclear Cell (PBMC) Isolation and Cell Culture

Peripheral blood mononuclear cells were isolated from heparinized blood by density gradient centrifugation (250 × *g* for 30 min at 21°C) with 3 mL of Ficoll-Hypaque^®^-1077 (Sigma, St. Louis, MO, USA). PBMCs were washed (1X PBS pH 7.4) and centrifuged (250 × *g* for 5 min at 21°C) three times. Next, 1 × 10^6^ cells per well were plated in 200 µL of RPMI medium supplemented with 10% fetal bovine serum, 100 U/mL penicillin, 100 µL streptomycin, and 2 nM l-glutamine (Sigma). Cell samples were incubated with 2 µL of phytohemmaglutinin (10 µg/mL; Sigma) per well in triplicates at 37°C for 24 h in a CO_2_ incubator (Sanyo Electric Biomedical Co., Ltd., Moriguchi, Osaka, Japan), and the supernatant was frozen at −80°C for chemokine analysis.

### Flow Cytometry

Chemokine concentration was measured using the BD™ CBA human chemokine kit according to the manufacturer’s instruction on a FACSVerse™ flow cytometer (Becton Dickinson, Sunnyvale, CA, USA). Approximately 300 events were acquired per capture bead. Analysis was performed using FCAP Array™ software (BD Biosciences, San Jose, CA, USA), and values are reported in picograms per milliliter.

### Fetal *RHD* Genotyping

Blood samples were transferred to 10 mL plastic tubes and centrifuged at 1,600 × *g* for 10 min. Plasma samples were transferred into a clean, labeled 10 mL plastic tube, centrifuged at 4,600 × *g* for 10 min, and supernatants were collected in 400 µL aliquots and stored at −20°C until further processing. DNA was extracted from 800 µL plasma samples using QIAmp Blood DNA Mini Kit (Qiagen, Valencia, CA, USA) according to the manufacturer’s instructions. DNA samples were amplified using TAQMAN real-time quantitative polymerase chain reaction. Primers and probes for *RHD* exons 4, 5, 7, 10, and for the *CCR5* gene were used. The controls used for fetal RhD genotyping were RhD-positive DNA, RhD-negative DNA, and RhD pseudogene DNA. Fetal *RHD* genotyping was performed according to the technique described by Finning (10).

A single reaction with a final volume of 25 µL was performed using 1X Universal PCR Master Mix (Applied Biosystems, Foster City, CA, USA), 200 nM of each primer, 100 nM of each probe, and 5 µL of DNA. To confirm the amount and quality of DNA in each sample, the *CCR5* gene was used as internal control for PCR in a separate reaction using 200 nM of each primer. Reactions were performed on a StepOnePlus™ Real-Time PCR machine (Applied Biosystems) with the following cycle conditions: 2 min at 50°C and 10 min at 95°C, followed by 45 cycles of 15 s at 95°C and 1 min at 60°C. *RHD* genotyping results were interpreted as RhD^+^ when at least 2/3 replicates in each of *RHD* exons 4, 5, 7, and 10 were positive (*C*_t_ < 42) and RhD^−^ (no PCR amplification) if none or 1/12 replicates for *RHD* exons had a *C*_t_ value < 42 and the remaining replicates had a *C*_t_ value > 42.

### Statistical Analysis

Statistical analysis was performed using GraphPad^®^ Prism 6 (GraphPad Software Inc., La Jolla, CA, USA). Data are presented as median. All scale variables were tested for normality using the D’Agostino-Pearson omnibus normality test. Quantitative variables were compared between groups using non-parametric Mann–Whitney *U* test. A *P*-Value <0.05 was considered significant.

## Results

### Population Demographics

Forty-six women (age range: 18–41 years) were included in this study. The biological and obstetric characteristics of the mothers are presented in Table 1. Parity ranged from one to eight, and few women were multiparous or had previous miscarriage, except in the RhD^−^/IAT^+^ group, in which almost one-third of the women had had at least one miscarriage.

**Table 1:** Population demographics.

Variables	RhD^−^/IAT^−^ (*N* = 30)	RhD^−^/IAT^+^ (*N* = 16)
**Age (years)**		
Min–Max	18–40	22–41
Median	30	33
**Parity**		
Nullipara	7	0
<2	19	6
≥2	4	10
Miscarriage	3	5
**Gestational age (weeks)**		
<28	13	6
≥28–36	12	6
≥36	5	4

*RhD^−^/IAT^−^, RhD-negative without maternal alloimmunization; RhD^−^/IAT^+^: RhD-negative with maternal alloimmunization; Min–Max, minimum and maximum*.

### Chemokines

**Figure 1:**
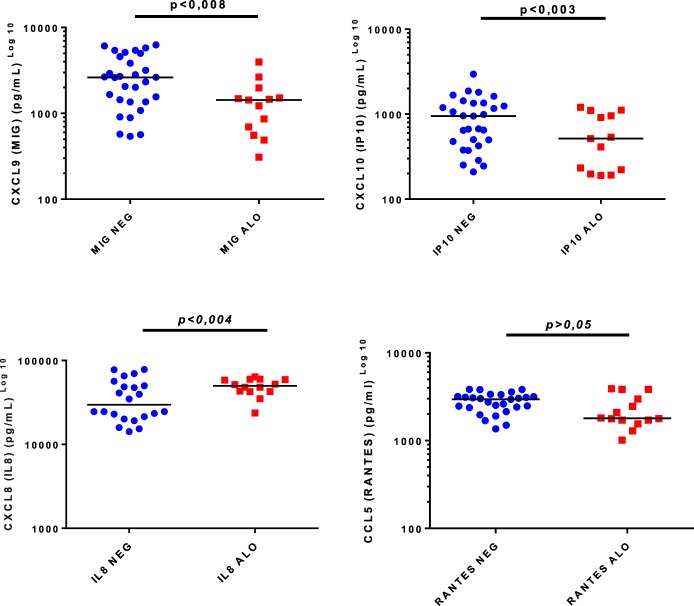
Chemokines and maternal RhD alloimmunization. Alo: RhD-negative women with maternal alloimmunization (IAT^+^); NEG: RhD-negative women without maternal alloimmunization (IAT^−^).

CXCL9 and CXCL10 levels were significantly higher in non-alloimmunized women (RhD^−^/IAT^−^), whereas CXCL8 levels were significantly higher in alloimmunized (RhD^−^/IAT^+^) pregnant women. No significant difference in CCL5 levels was detected between the groups (Table 2). In Figure 1 are presented chemokines according maternal RhD alloimmunization.

**Table 2:** Chemokines according to maternal RhD alloimmunization.

Chemokine median	RhD^−^/IAT^−^ (pg/mL)	RhD^−^/IAT^+^ (pg/mL)	***P
CXCL9 (MIG)	2,629	1,431	<0.008
CXCL10 (IP10)	948	515	<0.03
CXCL8 (IL-8)	29,655	49,763	<0.04
CCL5 (RANTES)	2,958	1,796	<0.07

*RhD^−^/IAT^−^, RhD-negative women without maternal alloimmunization; RhD^−^/IAT^+^, RhD-negative alloimmunized women*.

*Mann–Whitney U-test was used for all analysis*.

***P < 0.05 was considered significant.

### Fetal *RHD* Genotyping and Fetal and Neonatal Outcomes

Sixteen women had erythrocyte alloimmunization and were identified as anti-D (13/81%) and anti-D and -C (3/19%) by RBCA screening. Fetal *RHD* genotyping was performed for all women. Regarding alive fetus, 11 were male and 4 female. One mother had a stillbirth and her result could not be compared with the infant’s blood type. Concordance between fetal *RHD* genotype and newborn RhD status at delivery was as follows: 11 (74%) fetuses were RhD^+^ and four (26%) were RhD^−^. Cordocentesis was performed in seven fetuses at 28–32 weeks of gestation and was followed by IUT secondary to fetal anemia and/or fetal hydrops. One fetus had bradycardia after the IUT, and the pregnancy was therapeutically terminated by cesarean delivery. Two neonatal deaths from anti-D-positive mothers were recorded: one hydropic infant whose mother had gestational diabetes mellitus, polyhydramnios, and alloimmunization during pregnancy, and one newborn with congenital diaphragmatic hernia whose mother had premature rupture of membranes at 35 weeks of gestation. The stillbirth was secondary to fetal hydrops and his anti-D-positive mother had premature rupture of membranes at 25 weeks of gestation. All RhD^+^ babies born alive had jaundice and underwent phototherapy. Two anemic infants were delivered at private hospitals (2/16, 12.5%), and 14 (14/16, 87.5%) were delivered at IMIP.

## Discussion

Studies of *RHD* genotyping in pregnancy are essential to help understand the immune responses to fetal RBCs and the factors determining maternal alloimmunization. In this study, we present novel findings on the expression of the chemokines CXCL9 (MIG), CXCL10 (IP10), CXCL8 (IL-8), and CCL5 (RANTES) in cell culture supernatants in RhD-alloimmunized pregnant women. We found high levels of CXCL8 in alloimmunized RhD^−^ women, low levels of CXCL9 and CXCL10 in non-alloimmunized RhD^−^ women, and no difference in CCL5 levels between alloimmunized and non-alloimmunized RhD^−^ pregnant women.

### High CXCL8 Levels in Alloimmunized RhD Mothers

CXCL8 is a neutrophil chemoattractant (11) upregulated in inflammatory reactions that has been implicated in the influx of maternal immune cells into the decidua during labor (12) and in preeclampsia (13). Additionally, CXCL8 is a potent inducer of angiogenesis (14). In the case of maternal RhD alloimmunization, the immune response against protein antigens of fetal RBCs may result in fetal anemia (5, 6). In cases of fetal anemia and hemolytic disease of the fetus and newborn (HDFN), CXCL8 can induce placental neoangiogenesis and therefore increase maternal–fetal exchanges and blood supply in an attempt to compensate for fetal hypoxemia (14). This helps explain the significant increase in CXCL8 levels in pregnant women with RBC alloimmunization.

### Low CXCL9 and CXCL10 Levels in Alloimmunized RhD^−^ Pregnant Women

Both CXCL9 and CXCL10 are chemoattractants of effector T cells that bind to the CCR3 receptor (15, 16). CXCL10 specifically activates Th1 cells, and CXCL9 is a chemoattractant for Th1 and Th2 cells (15, 16). Thus, a reduction in CXCL10 and CXCL9 levels in alloimmunized pregnant women may be related to a protective mechanism involving reduced Th1 cell recruitment and activation, because Th1 cells are associated with adverse obstetric outcomes (17, 18). To date, no studies about CXCL10 and CXCL9 levels in RhD-alloimmunized pregnant women have been published. Future studies that analyze CXCL9 and CXCL10 plasma levels may help determine the numbers of Th1 and Th2 cells activated in maternal RhD alloimmunization.

### No Difference in CCL5 between Alloimmunized and Non-Alloimmunized RhD^−^ Pregnant Women

Once activated, platelets express chemokine receptors and several chemokines, including CCL5, CXCL4, CXCL5, and proangiogenic factors (19, 20). Platelets are involved in hemostasis and tissue repair and participate in innate and adaptive immune responses (20, 21). Thus, CCL5 could help maintain placental integrity in RhD^−^ pregnant women, reducing the chances of maternal–fetal cell trafficking and maternal exposure to fetal RhD RBC antigens. Moreover, CCL5 can induce apoptosis of T CD3^+^ cells and increase the frequency of regulatory T cells, which play an important role in maternal–fetal tolerance (20). Further studies comparing alloimmunized and non-alloimmunized RhD^+^ and RhD^−^ women could help elucidate the role of CCL5 during pregnancy and its involvement in maternal alloimmunization.

### Fetal *RHD* Genotyping in Alloimmunized Pregnant Women

Fetal *RHD* genotyping in maternal plasma was done only in alloimmunized (RhD^−^/IAT^+^) pregnant women for adequate clinical follow-up. In alloimmunized patients, knowledge that the fetus is *RHD*^−^ eliminates the need for intensive antenatal monitoring to predict and treat fetal anemia (22, 23). For alloimmunized women with an *RHD-*positive fetus, prenatal visits were intensified and fetal anemia was screened by obstetric color Doppler ultrasonography. MCA-PSV assessment is considered the primary technique to detect fetal anemia, and if a fetus is deemed at significant risk for severe fetal anemia, the patient must be referred to a center with expertise in invasive fetal therapy (24).

Fetal *RHD* genotyping is an accurate, non-invasive test based on the PCR technique using DNA isolated from maternal peripheral blood specimens (22, 23). The implementation of this method represents an important step toward the routine application of non-invasive fetal blood group diagnosis in alloimmunized pregnancies. PCR amplifies one or more regions of the fetal *RHD* gene in maternal plasma (22, 23). The accuracy of PCR for the detection of fetal *RHD*-specific DNA in maternal blood has been reported to be almost 99% (22, 23).

Unfortunately, fetal *RHD* genotyping is not easily accessible in Brazil, and only few health services offer such tests. However, implementation of *RHD* genotyping as a prenatal diagnostic test, at least in tertiary health care centers, could help identify pregnant women at risk for RhD alloimmunization and HDFN.

### Strengths and Limitations

Most studies analyzing the immunological aspects of maternal erythrocyte alloimmunization have been conducted in animal models, whereas the current study evaluated pregnant women. Moreover, this is the first study of maternal RhD alloimmunization to investigate chemokines that may be responsible for regulating leukocyte infiltration into the maternal decidua. However, further studies of serum chemokines and placenta tissue are required to identify the immune cells that infiltrate into the maternal decidua. Moreover, we analyzed fetal *RHD* genotype to better monitor fetuses at risk for HDFN. There was 100% concordance between real-time PCR genotyping and neonatal phenotype, demonstrating the excellent accuracy of the method. We could not compare chemokines from RhD-negative alloimmunized women according to (i) RhD status; (ii) fetal sex; (iii) fetal anemia; (iv) neonatal anemia because of the small number of subjects; however, this is a good objective for future research maybe in a multicentric study with more RhD-alloimmunized mothers and fetus involved.

## Ethics Statement

Clinical and laboratory protocols were approved by the Instituto de Medicina Integral Professor Fernando Figueira (IMIP) Research Ethics Committee (no. 3437-13). Written informed consent was obtained from all participants.

## Author Contributions

JS: antenatal and postnatal care; RhD fetal genotyping; cell acquisition, flow cytometry, statistical analysis, interpretation of data, wrote and approved the final draft. TG and AB: collect peripheral blood, PBMC isolation and cell culture, cell acquisition, flow cytometry, wrote and approved the final draft. MM: PBMC isolation and cell culture, statistical, analysis and interpretation of data, wrote and approved the final draft. IC: antenatal and postnatal care, statistical analysis, interpretation of data, wrote and approved the final draft. MO: RhD fetal genotyping, blood type and indirect antigammaglobulin test analysis, interpretation of data, wrote and approved the final draft. LT: RhD fetal genotyping; chemokines analysis, statistical analysis, and interpretation of data, wrote and approved the final draft.

## Conflict of Interest Statement

The authors declare that the research was conducted in the absence of any commercial or financial relationships that could be construed as a potential conflict of interest.
